# Immune-mediated remodelling of tissue architecture in Post-Kala-Azar Dermal Leishmaniasis (PKDL)

**DOI:** 10.1371/journal.pntd.0014798

**Published:** 2026-10-16

**Authors:** Deep Goswami, Shriya Saha, Madhurima Roy, Ebrahim Mohammad, Ratnaprabha Maji, Ritika Sengupta, Shilpa Sengupta, Sutopa Roy, Surya Jyati Chaudhuri, Nilay Kanti Das, Bidhan Chakraborty, Uttara Chatterjee, Mitali Chatterjee

**Affiliations:** 1 Department of Pharmacology, Institute of Postgraduate Medical Education and Research, Kolkata, India; 2 Department of Pathology, Institute of Postgraduate Medical Education and Research, Kolkata, India; 3 Department of Pathology, Nil Ratan Sircar Medical College and Hospital, Kolkata, India; 4 Department of Microbiology, Sarat Chandra Chattopadhyay Government Medical College and Hospital, Howrah, India; 5 Department of Dermatology, College of Medicine and Sagore Dutta Hospital, Kolkata, India; 6 Multidisciplinary Research Unit (MRU), Institute of Postgraduate Medical Education and Research, Kolkata, India; CSIR-Indian Institute of Chemical Biology, INDIA

## Abstract

**Background:**

Post-Kala-Azar Dermal Leishmaniasis (PKDL), a dermal sequel of apparently cured visceral leishmaniasis (VL), presents with macular or polymorphic lesions. A relationship, if any, between immune cell infiltration and cytokines potentially causing tissue-remodelling remains poorly defined, and was the focus of this study.

**Methodology/principal findings:**

Skin biopsies along with plasma from PKDL patients and healthy controls were analyzed. Tissue sections were assessed for immune cell subsets (CD8 ⁺ T cells, CD68 ⁺ macrophages, CD20 ⁺ B cells, CD66b⁺ neutrophils), associated cytokines/chemokines, adhesion molecules (E-cadherin, Intercellular Adhesion Molecule-1), extracellular matrix (ECM) proteins (fibronectin, collagen I/IV, laminin), matrix metalloproteinase 9 (MMP9) using immuno-histochemistry/fluorescence, while circulating cytokines/chemokines, and MMP9 were quantified using multiplex assays/ELISA. PKDL lesions exhibited increased cell infiltration, monocytes/macrophages>CD8 ⁺ T cells>> neutrophils >B cells. E-cadherin was significantly downregulated, especially in macular lesions, whereas ICAM-1 was elevated. In both PKDL forms, ECM components were significantly reduced, and correlated inversely with immune cell infiltration. The raised levels of circulating chemokines (CCL2, CCL3, CCL4, CCL5, CXCL9, CXCL10, IL-8) positively correlated with their corresponding immune populations; additionally, circulating pro- (TNF-α, IFN-γ, IL-6, IL-1β) inflammatory cytokines and IL-4 was elevated. Additionally, lesional TNF-α, IFN-γ and MMP9 was elevated, and correlated inversely with E-cadherin and ECM proteins.

**Conclusions/significance:**

In PKDL lesions, there was an elevated presence of monocytes-macrophages/CD8^+^ T cells, B cells and neutrophils; the increased presence of their respective chemokines endorsed their homing to lesional sites. Furthermore, the increased presence of dermal MMP9, TNF-α and IFN-γ possibly facilitated the breakdown of E-cadherin and ECM components, which could cause a site-specific loss of melanocytes, and contribute to the hypopigmentation associated with PKDL.

## Author summary

Post Kala-Azar Dermal Leishmaniasis (PKDL), caused by the digenetic parasite *Leishmania donovani*, and transmitted by the female sand fly presents as a chronic dermal condition in a subset of apparently cured patients with visceral leishmaniasis. PKDL occurs mainly in East Africa and South Asia in 20–50% and 2.5-20% cases of VL respectively. In South Asia, PKDL presents with nodules, papules and/or macules (polymorphic form) or hypopigmented macules (macular form). Owing to its relatively benign, non-lethal nature, patients with PKDL have a poor health seeking behavior and thereby become mobile reservoirs promoting disease transmission. As patients present with hypopigmented lesions, akin to Leprosy, another NTD, they face stigmatization and ostracization. In the absence of an animal model for PKDL, factors involved in the pathogenesis of this hypopigmentation remain poorly defined. Skin biopsies and plasma from patients with polymorphic and macular PKDL were evaluated to understand whether the infiltration of immune cells had any impact on the skin architecture and vice versa. PKDL lesions were characterized by dense immune cell infiltration driven by elevated chemokines and adhesion molecules, particularly ICAM-1. Concomitantly, there is a profound disruption of the epidermal dermal junction, characterized by reduced E-cadherin along with degradation of key extracellular matrix components, fibronectin, collagen I, collagen IV, and laminin, that singly and/or collectively facilitated immune cell infiltration into lesional sites of PKDL, thus perpetuating the disease. These changes are strongly associated with increased levels of pro-inflammatory cytokines (Tumor necrosis factor-alpha and Interferon-gamma) along with matrix metalloproteinase-9 (MMP9), both locally and systemically. As MMP9 is known to correlate negatively with structural proteins essential for melanocyte adhesion, it provides a possible mechanistic link between inflammation, tissue damage, and hypopigmentation. Together, these findings indicate that MMP9 mediated matrix remodelling is a possible central pathway driving skin pathology and hypopigmentation in PKDL.

## Introduction

*Leishmania*, despite being a single genus, includes over 20 species, accounting for diverse clinical manifestations, ranging from localized cutaneous leishmaniasis (CL), mucocutaneous leishmaniasis (MCL), diffuse cutaneous leishmaniasis (DCL) to severe, fatal if untreated visceral leishmaniasis (VL). Post Kala-azar Dermal Leishmaniasis (PKDL), a dermal sequel of VL, occurs in 2.5-20% of treated VL cases in South Asia and 20–50% in Sudan [1]. In India, PKDL presents with polymorphic (papules, nodules, macules) or macular (hypopigmented patches) forms [2,3].

The human skin functions as a structural and immunological barrier, composed of epithelial cells, majorly keratinocytes followed by melanocytes, that are held together with the basement membrane by cell adhesion molecules and extracellular matrix components, thereby collectively preserving tissue integrity and immune defense [4]. The extracellular matrix (ECM) is composed of structural proteins such as collagen, elastin, laminin, and fibronectin that fortify the skin’s mechanical strength and elasticity, as also provide a critical scaffold for cellular adhesion, migration, and tissue repair [5,6].

In the basal layer of the epidermis, E-cadherin, a calcium-dependent adhesion protein, is critical for maintaining adhesion between keratinocytes and melanocytes, thereby supporting epidermal homeostasis and pigmentation, [7]. In the pathogenesis of hypopigmentation associated with vitiligo, it has been proposed that disruption of E-cadherin can lead to detachment of melanocytes [8]. Additionally, disruption of the ECM components, such as fibronectin and laminin, which are largely driven by elevated levels of TNF-α and IFN-γ, can induce the matrix metalloproteinase-9 (MMP9), and augment tissue damage [8,9]. The pathogenesis of CL is associated with degradation of ECM components such as collagen I, IV and fibronectin [10,11]. This tissue remodelling is accompanied by an elevated expression of ICAM-1, that facilitates increased cellular infiltration [12] and along with an enhanced production of matrix metalloproteinases (MMPs), particularly MMP2 and MMP9, mediates breakdown of the ECM resulting in ulceration [13,14]. This was substantiated by the use of MMP inhibitors, e.g., doxycycline, which minimized tissue damage and enhanced healing [15].

In the context of PKDL, studies have focused on characterization of the immune cell infiltrate and the inflammatory milieu [16–18], wherein a reduced expression of collagen I, increased levels of circulating pro-inflammatory cytokines and elevated level of MMP9 have been demonstrated, locally and systemically [16,19]. However, a pertinent yet unanswered question is the contributory role, if any, of these elevated pro-inflammatory cytokines and MMP9 in altering the tissue microarchitecture, and accounting for the hypopigmentation observed in PKDL. The primary objective of this study was to investigate whether there are alterations in the tissue microarchitecture of skin lesions of patients with PKDL, and if so, whether these changes are associated with clinical features of PKDL.

## Results

### Study population

The study population (n = 36) included a comparable proportion of polymorphic (*n* = 18) and macular (*n* = 18) cases of PKDL, to reflect the present demographic scenario reported in West Bengal, India [3]. Polymorphic cases of PKDL presented with papules, nodules and/or macules, whereas the macular cases of PKDL presented only with macules/hypopigmentation. Majority of the polymorphic form presented with all types of lesions, i.e., macules, papules and nodules. Majority of the cases (n = 30/36, 83.3%) were sourced by active surveillance, and a male preponderance was evident in the polymorphic form, whereas the macular form had no gender bias [Table 1]. Majority (34/36, 94%) gave a prior history of VL, but 7/36 (19.4%) were unable to recall specific treatment received for VL. Amongst the analysable 29/36 cases, during their episode of VL, they received sodium antimony gluconate (SAG, *n* = 20/29, 69%, polymorphic, n = 12; macular, n = 8) or Miltefosine (5/29, 17%; polymorphic, n = 2; macular, n = 3) or Liposomal Amphotericin B (4/29, 13.8%; polymorphic, n = 1; macular, n = 3). The polymorphic cases as compared to macular cases presented with a two-fold higher disease duration and a 2.2-fold longer interval between VL and onset of PKDL. The parasite load as quantified by qPCR, was 1.67-fold higher in the polymorphic form *vis-à-vis* the macular form [Table 1]. PKDL is a systemic disease and Moulik et al., [20] have demonstrated in three randomly selected cases of polymorphic PKDL, that the parasite load in a PKDL patient, irrespective of the type of lesion, is comparable. In the polymorphic PKDL cases, a biopsy was obtained from a nodular lesion, whereas in the macular PKDL group, biopsy was obtained from a hypopigmented lesion [20]. It was challenging to obtain skin biopsies from healthy volunteers, being an invasive and relatively painful procedure. Instead preputial skin was used which in previous studies has been reported to have a cellular infiltrate comparable to healthy skin [16,21–23], and accordingly served as control tissue. To corroborate this, skin tissues were collected from (i) individuals undergoing plastic surgery and (ii) preputial skin, and their immune cell infiltration was quantified. There were no differences in the cellular infiltration, both in terms of number and composition (S1 Fig) In case of blood, the controls were age- and gender-matched healthy volunteers. All procedures were performed following approval from the Institutional Ethics Committee after obtaining informed consent from participants or their legally accepted representative.

**Table 1:** Study population.

Characteristics	Patients with PKDL		Healthy controls	
	Polymorphic (n = 18)	Macular (n = 18)	Skin biopsy (n = 12)	Peripheral blood (n = 11)
Age (years)^a^	30.0 (23.0-45.0)	28.0 (17.3-47.0)	3.0 (2.1-4.0)	29.0 (27.0-35.0)
Gender (M: F)	17:1	8:10	Only males	4:7
Disease duration (years)^a^	4.00 (1.7-8.6)	2.00 (1.0-5.3)	NA	NA
Interval between VL and PKDL (years)^a^	9.0 (3.3-13.5)	4.0 (0.5-14.0)	NA	NA
Parasite load (parasites/ μg genomic DNA)^a^	10088.0 (760.5-801726.0)	6028.0 (654.0-34062.0)	NA	NA

Skin biopsies and peripheral blood was obtained from cases of PKDL and healthy controls as described in Materials and methods. F, female; M, male; NA, not applicable; PKDL, post Kala-azar Dermal Leishmaniasis; VL, visceral leishmaniasis. ^a^values are expressed as median (IQR).

### Status of the cellular infiltrate in patients with PKDL

Overall, the cellular infiltration in PKDL cases [215.0(180.3-249.0) cells/field] was substantially higher than healthy controls [31.5(24.0-41.7) cells/field, p < 0.001, Fig 1A], the density in polymorphic and macular forms being comparable, median (IQR) being [250.0(190.0-290.0) cells/field] vs. [200.0(166.0-225.0) cells/field] respectively [Fig 1A]. In both forms, the infiltrate was restricted to the dermis, with the polymorphic form showing a diffuse distribution, whereas in the macular form, it was patchy, and in clusters mainly within the reticular dermis, aligning with previous studies [3,16]. This increased cellular infiltrate exhibited a positive correlation with parasite load (r = 0.58; p < 0.05) and corroborated with previous studies [24]

**Fig 1:**
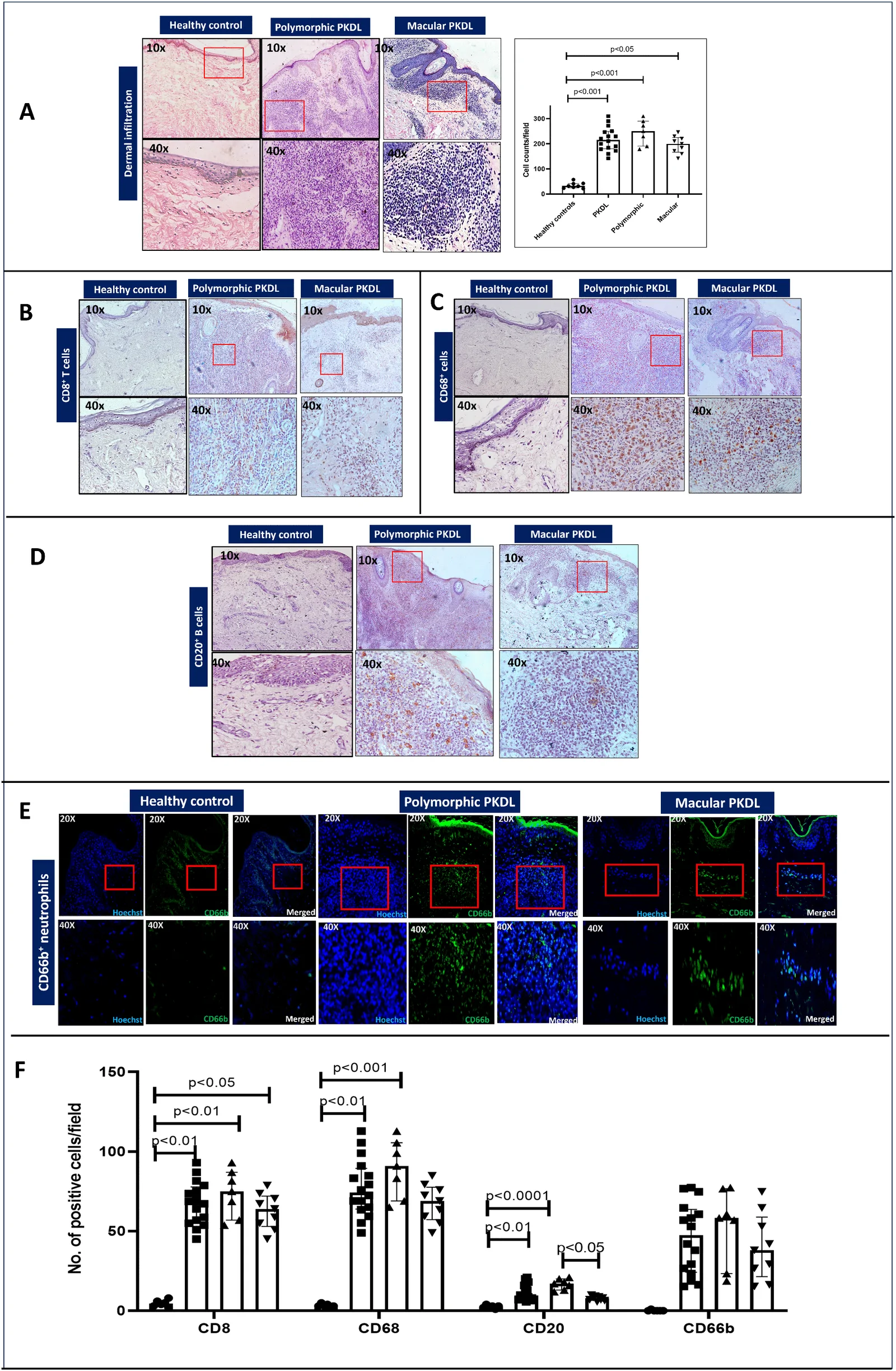
Status of the cellular infiltrate in patients with PKDL. **A:** Representative immunohistochemical profiles of the cellular infiltrate in dermal biopsies of a healthy control, patient with polymorphic or macular PKDL, 10x and 40x magnification, along with bar scatter plots indicating the cellular infiltrate in healthy controls (●, n = 6) and patients with PKDL (■, n = 16), either polymorphic (▲, n = 7) or macular (▼, n = 9) PKDL; each horizontal bar represents the median (IQR). **B-E:** Representative immunohistochemical/immunofluorescence profiles of CD8^+^ T cells (**b**), CD68^+^ monocytes-macrophages (**c**) CD20^+^ B cells (**d**) and CD66b^+^ neutrophils (**e**) in dermal biopsies of a healthy control, patient with polymorphic or macular PKDL, 10x and 40x magnification. **F:** Bar scatter plots indicating the status of CD8^+^ T cells, CD68^+^ monocytes-macrophages, CD20^+^ B cells and CD66b^+^ neutrophils in healthy controls (●, n = 6), patients with PKDL (■, n = 16), either polymorphic (▲, n = 7) or macular (▼, n = 9) PKDL; each horizontal bar represents the median (IQR).

#### Status of CD8^+^ T cells in patients with PKDL.

In PKDL, the infiltration of CD8^+^ T cells was higher than healthy controls [68.0(55.5-77.8) vs. 4.5(2.8-6.5) cells/field, p < 0.01] [Fig 1B and 1F], but their density was comparable between polymorphic and macular PKDL [Fig 1B and 1F], being 75.0(57.0-87.0) and 64.0(53.0-72.0) cells/field respectively [Fig 1B and 1F]. In 9/16 (56%), the cells were predominantly restricted to the upper dermis, and positively correlated with the parasite load (r = 0.61; p < 0.05), as previously reported [24].

#### Status of CD68^+^ monocytes-macrophages in patients with PKDL.

In alignment with previous studies [3,25], this study corroborated that CD68^+^ cells are located just beneath the papillary dermis. The proportion of CD68^+^ cells in healthy controls [3.0 (2.7-4.3) cells/field] [Fig 1C and 1F] increased by 24.6-fold in PKDL cases, being 74.0(64.0-89.4) cells/field, p < 0.01, and positively correlated with the parasite load (r = 0.54; p < 0.05). On a lesional basis, the proportion of infiltration was 30.3-fold higher in the polymorphic group, being [91.0(69.0-105.5) cells/field, Fig 1C and 1F]. Similarly, in the macular form there was a 23-fold enhancement, 69.0 (57.2-77.6) cells/field [Fig 1C and 1F].

#### Status of CD20^+^ B cells in patients with PKDL.

The proportion of CD20^+^ B cells, as compared to healthy controls [3.0(1.7-3.2) cells/field, Fig 1D] increased by 3.2-fold being [9.5(7.2-16.7) cells/field, p < 0.01]. On a lesional basis, the infiltration was 5.6-fold higher in the polymorphic group [17.0(13.0-20.0) cells/field, Fig 1D], whereas the macular form showed a 2.6-fold enhancement [8.0 (6.7-9) cells/field, Fig 1D and 1F].

#### Status of CD66b^+^ neutrophils in patients with PKDL.

The CD66b^+^ neutrophils were diffusely distributed in the reticular dermis, their proportion being significantly higher in PKDL cases [47.5 (24.1-63.6) cells/field], as compared to healthy controls, where they were minimally present/absent [Fig 1E]. On a lesional basis, the infiltration in polymorphic and macular forms was comparable, being 58.3(23.3-76.6) and 38.0(21.5-58.8) cells/field respectively [Fig 1E and 1F cells/field].

### Status of circulating chemokines in patients with PKDL

Chemokines are small, secreted signaling proteins that play a vital role in directing immune cell migration along concentration gradients toward sites of inflammation or homeostatic tissues [26]. CCL2, a major chemoattractant for CD68 ⁺ monocytes/macrophages, was upregulated by 17.8-fold, and positively correlated with dermal monocyte/macrophage infiltration [Table 2]. Similarly, chemokines CXCL9, CXCL10, CCL3, CCL4, and CCL5 in circulation were significantly elevated, and positively associated with CD8 ⁺ T-cell infiltration, while CXCL9 and CXCL10 levels showed a positive correlation with CD20 ⁺ B cells. In addition, CXCL8, a potent chemoattractant for CD66b⁺ neutrophils, exhibited a 940-fold increase, and strongly correlated with neutrophil infiltration [Table 2].

**Table 2:** Status of circulating levels of chemo-attractants and correlation with cellular infiltrate.

Chemokines	PKDL (pg/ml),[n = 16]	Healthy controls (pg/ml),[n = 9]	Fold increase	p value	Correlation with immune cells [n = 16]
**CCL2**	277.1(213.2-1215.0)	15.5(10.4-19.5)	17.87	p < 0.0001	*r = 0.49
**CXCL9**	2216.0(982.7-30766.0)	825.3(593.8-1557.0)	2.7	p < 0.05	^#^r = 0.68; p < 0.01 ^@^r = 0.43
**CXCL10**	8911.0(3012.0-25469.0)	2229.0(1906.0-2985.0)	4.0	p < 0.01	^#^r = 0.69; p < 0.05 ^@^r = 0.66; p < 0.05
**CCL3**	87.6(13.6-638.8)	1.9(1.0-2.2)	46.1	p < 0.001	^#^r = 0.61; p < 0.05
**CCL4**	1084.0(916.9-2027.0)	427.4(372.1-555.1)	2.5	p < 0.001	^#^r = 0.48
**CCL5**	21602.0(15706.0-29804.0)	5978.0(4713.0-9696.0)	3.6	p < 0.0001	^#^r = 0.35
**CXCL8**	33768.0(17436.0-64446.0)	35.9(12.9-422.4)	940.6	p < 0.0001	^$^r = 0.57; p < 0.05

The circulating levels of chemo-attractants and their correlation with infiltrating immune cells (*CD68^+^ monocytes-macrophages; ^#^CD8^+^ T cells; ^@^CD20^+^ B cells; ^$^CD66b^+^ neutrophils) as described in materials and methods. Values are stated in median (IQR).

### Lesional status of E-Cadherin in patients with PKDL

E-Cadherin, a key cell adhesion molecule, functions as a bridge between keratinocytes and melanocytes in the epidermis [8]. While alterations in its expression have been studied in vitiligo, a prototype example of hypopigmentation, its role and expression in PKDL, which also exhibits hypopigmentation, remains unclear. Accordingly, the % E-Cadherin positivity was evaluated in PKDL cases, wherein a 2.2-fold decrease was evident *vis-à-vis* healthy controls, being 40.3 (33.0-43.2) vs. 89.0 (85.0-95.0) percent positive cells/field, p < 0.01 [Fig 2A]. At lesional sites, as compared to skin of healthy controls, decrease in the polymorphic and macular form was similar, being [41.8 (32.0-51.2), p < 0.05] and [39.3 (34.3-43.3) percent positive cells/field, p < 0.01] respectively [Fig 2A].

**Fig 2:**
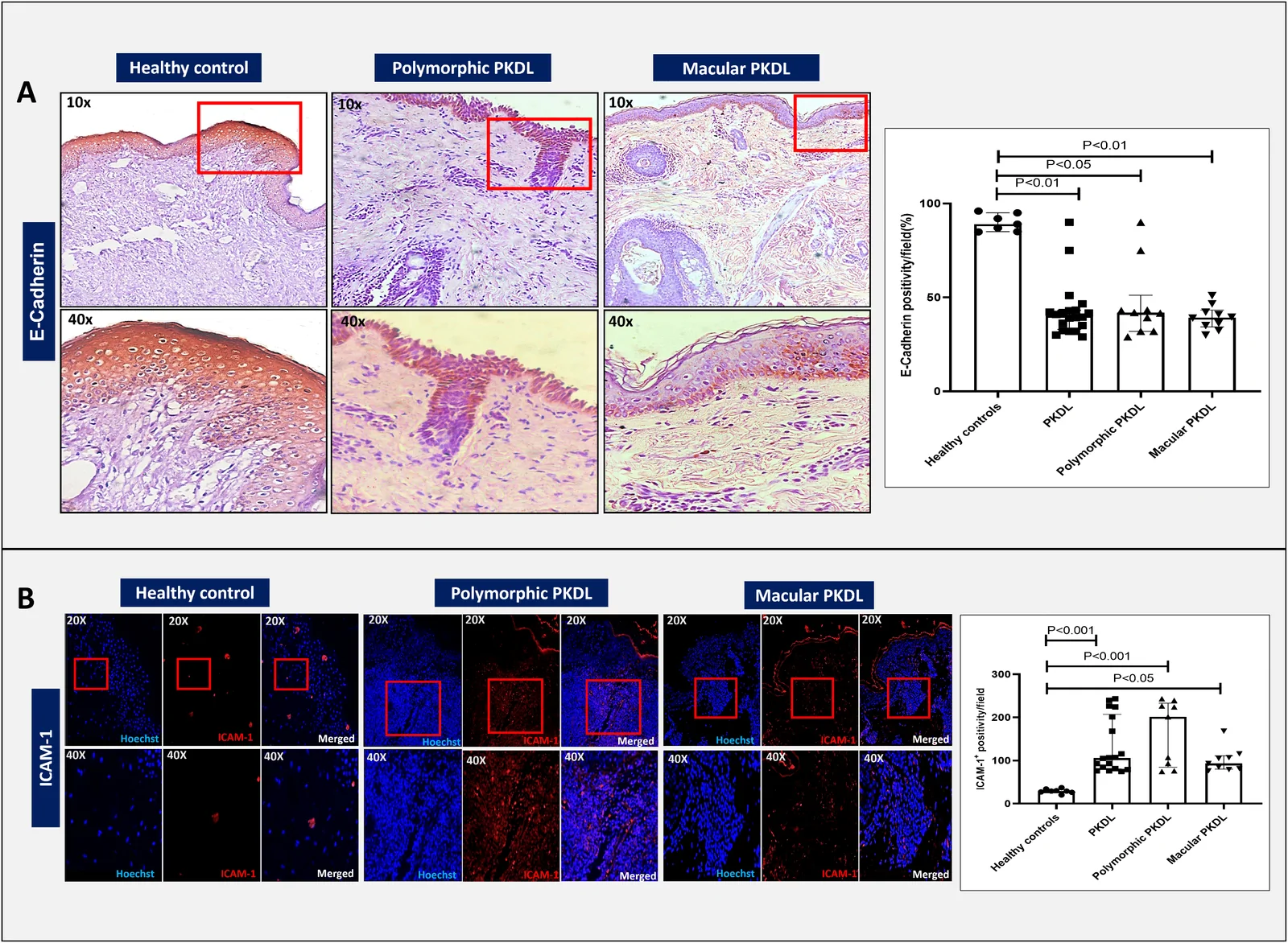
Status of cell adhesion molecules in patients with PKDL. **A:** Representative immunohistochemical profiles of E-Cadherin from dermal biopsies of a healthy control, patient with polymorphic or macular PKDL, 10x and 40x magnification along with bar scatter plots indicating the status of E-Cadherin in healthy controls (●, n = 7), patients with PKDL (■, n = 20), either polymorphic (▲, n = 10) or macular (▼, n = 10) PKDL; each horizontal bar represents the median (IQR). **B:** Representative immunofluorescence profiles of ICAM-1 from dermal biopsies of a healthy control, patient with polymorphic or macular PKDL, 20x and 40x magnification along with bar scatter plots showing the status of ICAM-1 in healthy controls (●, n = 8), patients with PKDL (■, n = 18), either polymorphic (▲, n = 9) or macular (▼, n = 9) PKDL; each horizontal bar represents the median (IQR).

### Lesional status of Intercellular Adhesion Molecule-1 (ICAM-1) of patients with PKDL

ICAM-1, a cell surface glycoprotein primarily involved in facilitating leukocyte recruitment to sites of inflammation, is expressed on vascular endothelial cells, immune cells, particularly lymphocytes and macrophages [27]. Although alterations in their expression patterns have been studied in vitiligo, their role and expression in PKDL, which also features hypopigmentation, remains to be delineated. In PKDL cases, the ICAM-1 positivity as compared to healthy controls increased by 3.6-fold being [105.7(80.7-207.1) vs. [29.0(26.3-33.0) fluorescence positive cells/field] p < 0.001, Fig 2B]. On a lesional basis, as compared to healthy skin, the proportion of ICAM-1 positivity was raised in both forms, being 7- and 3-fold higher in the polymorphic and macular group respectively, being [201.3(84.5-232.2), p < 0.001], and [93.0(80.2-111.2) fluorescence positive cells/field, p < 0.01] respectively [Fig 2B]. This enhanced expression of ICAM-1 correlated positively with the overall dermal infiltrate, along with the individual cellular components [Table 3].

**Table 3:** Correlation between extracellular matrix components and the cellular infiltrate in patients with PKDL.

ECM protein components [n = 16]	Total immune cells and its components [n = 16]				
	Cellular infiltrate	CD8 ^+^ T cells	CD68 ^+^ monocytes-macrophages	CD20 ^+^ B cells	CD66b^+^ neutrophils
**ICAM-1**	r = 0.64; p < 0.05	r= 0.72; p<0.05	r = 0.44	r = 0.73; p < 0.01	r = 0.57
**Fibronectin**	r= -0.65; p<0.05	r = -0.52	r = -0.62; p < 0.05	r= -0.61; p<0.05	r = -0.45
**Collagen I**	r= -0.58; p<0.05	r = -0.53	r = -0.5	r = -0.25	r = -0.68; p < 0.05
**Collagen IV**	r= -0.76; p<0.01	r= -0.74; p<0.05	r= -0.7; p<0.05	r = -0.37	r = -0.61
**Laminin**	r = -0.6; p < 0.05	r = -0.53	r = -0.55	r = -0.31	r = -0.47

### Lesional status of fibronectin in patients with PKDL

Fibronectin, an essential extracellular matrix glycoprotein, plays a critical role in skin tissue by mediating cell adhesion, migration, and wound healing [28], and its disruption impedes melanocyte adhesion and extracellular matrix remodelling, leading to a loss in pigmentation [7,9]. The expression of fibronectin in PKDL cases decreased significantly by 3.2-fold as compared to healthy controls, being [43.8 (41.3-46.8) vs. [140.7 (98.6-164.0) fluorescence positive cells/field, p < 0.001] [Fig 3A]. On a lesional basis, both polymorphic and macular PKDL forms, showed a 3.2- and 3.1-fold reduction, being [43.4 (39.3-46.6), p < 0.01] and [45.0 (40.7-47.9) fluorescence positive cells/field, p < 0.01] respectively [Fig 3A]. Notably, this decreased expression of fibronectin negatively correlated with the dermal infiltrate [Table 3].

**Fig 3:**
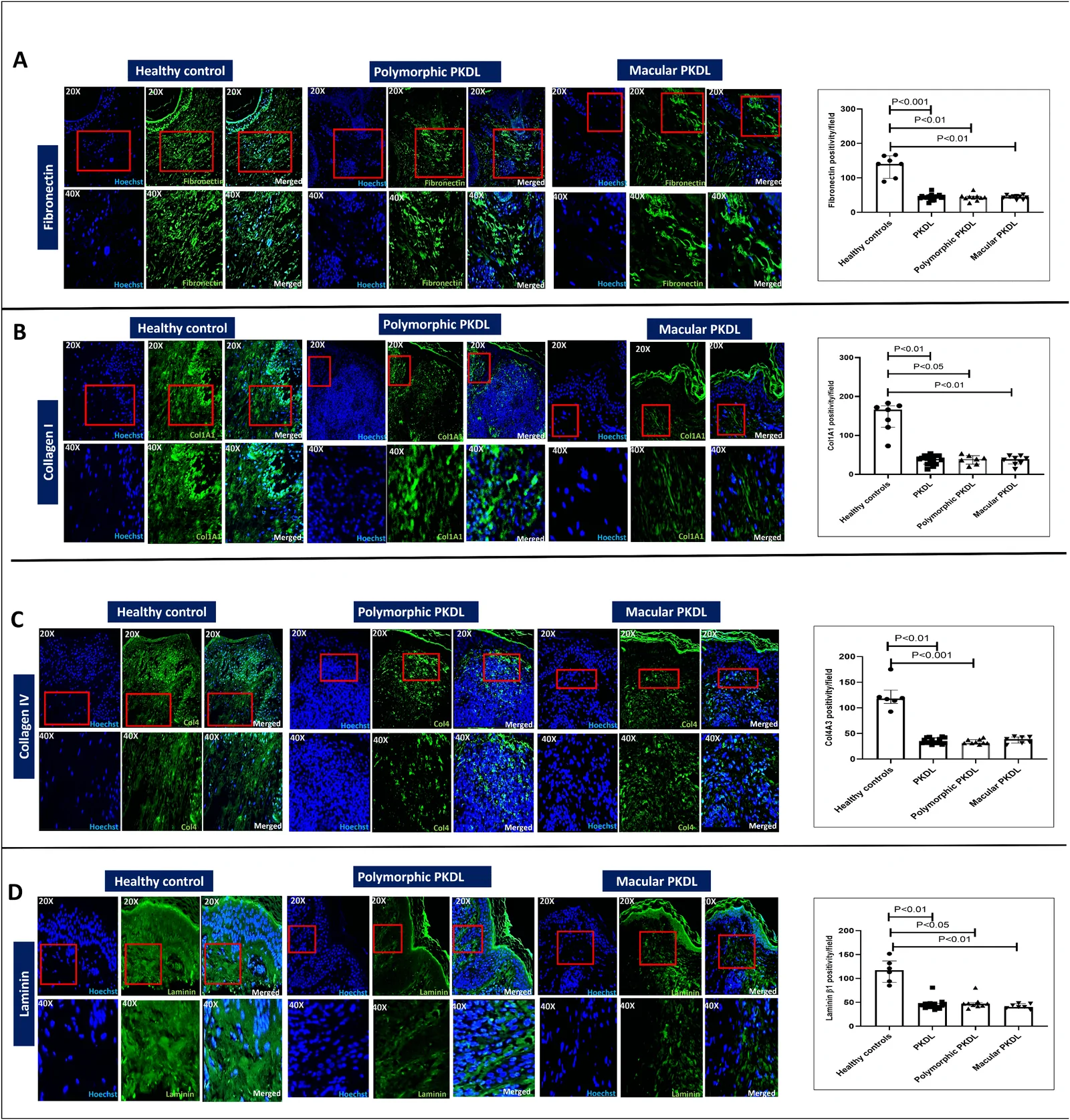
Status of extracellular matrix components in patients with PKDL. **A:** Representative immunofluorescence profiles of fibronectin in dermal biopsies of a healthy control, patient with polymorphic or macular PKDL (20x and 40x magnification). Bar scatter plots showing the status of fibronectin in healthy controls (●, n = 7), patients with PKDL (■, n = 20), either polymorphic (▲, n = 10) or macular (▼, n = 10) as fluorescence positivity/field; each horizontal bar represents the median (IQR). **B:** Representative immunofluorescence profiles of Collagen I expression in dermal biopsies of a healthy control, patient with polymorphic or macular PKDL (20x and 40x magnification). Bar scatter plots quantifying the expression of Col1 in healthy controls (●, n = 7), patients with PKDL (■, n = 15), either polymorphic (▲, n = 7) or macular (▼, n = 8) PKDL as fluorescence positivity/field; each horizontal bar represents the median (IQR). **C:** Representative immunofluorescence profiles of Collagen IV expression in dermal biopsies of a healthy control, patient with polymorphic or macular PKDL (20x and 40x magnification). Bar scatter plots quantifying the status of Col4 in healthy controls (●, n = 6), patients with PKDL (■, n = 16), either polymorphic (▲, n = 9) or macular (▼, n = 7) PKDL as fluorescence positivity/field; each horizontal bar represents the median (IQR). **D:** Representative immunofluorescence profiles of laminin expression from dermal biopsies of a healthy control, patient with polymorphic or macular PKDL (40x magnification) along with bar scatter plots showing the status of laminin expression in healthy controls (●, n = 6), patients with PKDL (■, n = 16), either polymorphic (▲, n = 9) or macular (▼, n = 7) PKDL as fluorescence positivity/field; each horizontal bar represents the median (IQR).

### Lesional status of Collagen I and IV in patients with PKDL

Collagen I and IV are key components of the skin’s extracellular matrix, with type I providing dermal tensile strength, and type IV supporting basement membrane adhesion and filtration, and its disruption is associated with hypopigmentation. In PKDL, as compared to healthy controls, Collagen I positivity decreased substantially by 4.2-fold [39.3 (26.0-47.7) vs. 166.0 (121.0-176.0) fluorescence positive cells/field, p < 0.01] [Fig 3B]; this decline was comparable in both forms, being 4.2- and 4.3-fold in the polymorphic and macular form, 39.3 (26.0-48.0), p < 0.05] and 38.8 (26.7-46.2) fluorescence positive cells/field, p < 0.01 respectively, [Fig 3B].

Similarly, collagen IV in PKDL lesions as compared to healthy skin decreased by 3.6-fold being [32.7 (31.0-41.5) vs. 118.0 (108.6-134.5) fluorescence positive cells/field, p < 0.01] [Fig 3C]. The lesion-wise reduction in the polymorphic and macular form was 3.7- and 3.0-fold, being 31.5 (31.0-38.0), p < 0.001] and 39.0 (31.3-43.0) fluorescence positive cells/field] respectively and correlated negatively with the total cellular infiltrate [Table 3]. [Fig 3C]. Additionally, the proportion of collagen was assessed in preputial skin and skin from healthy adults. A quantitative analysis of collagen content revealed no significant differences (S2 Fig).

### Lesional status of laminin in patients with PKDL

Laminin, a crucial component of the basement membrane in skin tissue, facilitates cell adhesion, migration, and structural integrity. In vitiligo, disorganization of the basement membrane, includes an altered distribution of laminin, impaired melanocyte adhesion, collectively contributing to depigmentation [29,30]. In PKDL, the laminin positivity as compared to healthy controls was decreased by 2.6-fold being [44.0(40.1-47.6) vs. 117.0 (91.7-136.6)] fluorescence positive cells/ field, p < 0.01, [Fig 3D]. On a lesional basis, the reduction of laminin in polymorphic and macular PKDL was 2.5 and 3.0-fold being [47.0(42.3-49.5), p < 0.05 and [40.3 (38.0-47.3)] fluorescence positive cells/field, p < 0.01 respectively [Fig 3D], and correlated negatively with the cellular infiltrate [Table 3].

### Lesional status of cytokines in patients with PKDL

Pro-inflammatory cytokines like TNF-α and IFN-γ contribute to hypopigmentation by inhibiting melanogenesis and inducing apoptosis of melanocytes [31]. Previous studies have established that the circulating levels of pro-inflammatory cytokines are raised in PKDL, and our findings were in concordance [Fig 4A and Table 4]. However, information pertaining to their dermal status was limited [19,32].

**Table 4:** Status of circulating levels of cytokines in PKDL.

Chemokines	PKDL (pg/ml), [n=16]	Healthy controls (pg/ml) [n = 9]	Fold change	p value
**TNF-α**	81.1 (43.7-131.9)	11.5 (9.3-17.5)	7.05	p < 0.01
**IFN-γ**	108.9 (53.1-221.3)	34.2 (26.2-49.2)	3.18	p < 0.001
**IL-1β**	22.9 (12.1-88.9)	3.7 (2.1-9.6)	6.18	p < 0.001
**IL-6**	50.7 (9.2-755.3)	5.2 (1.8-6.7)	9.75	p < 0.001
**IL-4**	28.3 (12.1-80.3)	11.8 (8.7-23.4)	2.39	NS

The circulating levels of cytokines were measured as described in Materials and methods. Values are stated in median(IQR). NS = not significant.

**Fig 4:**
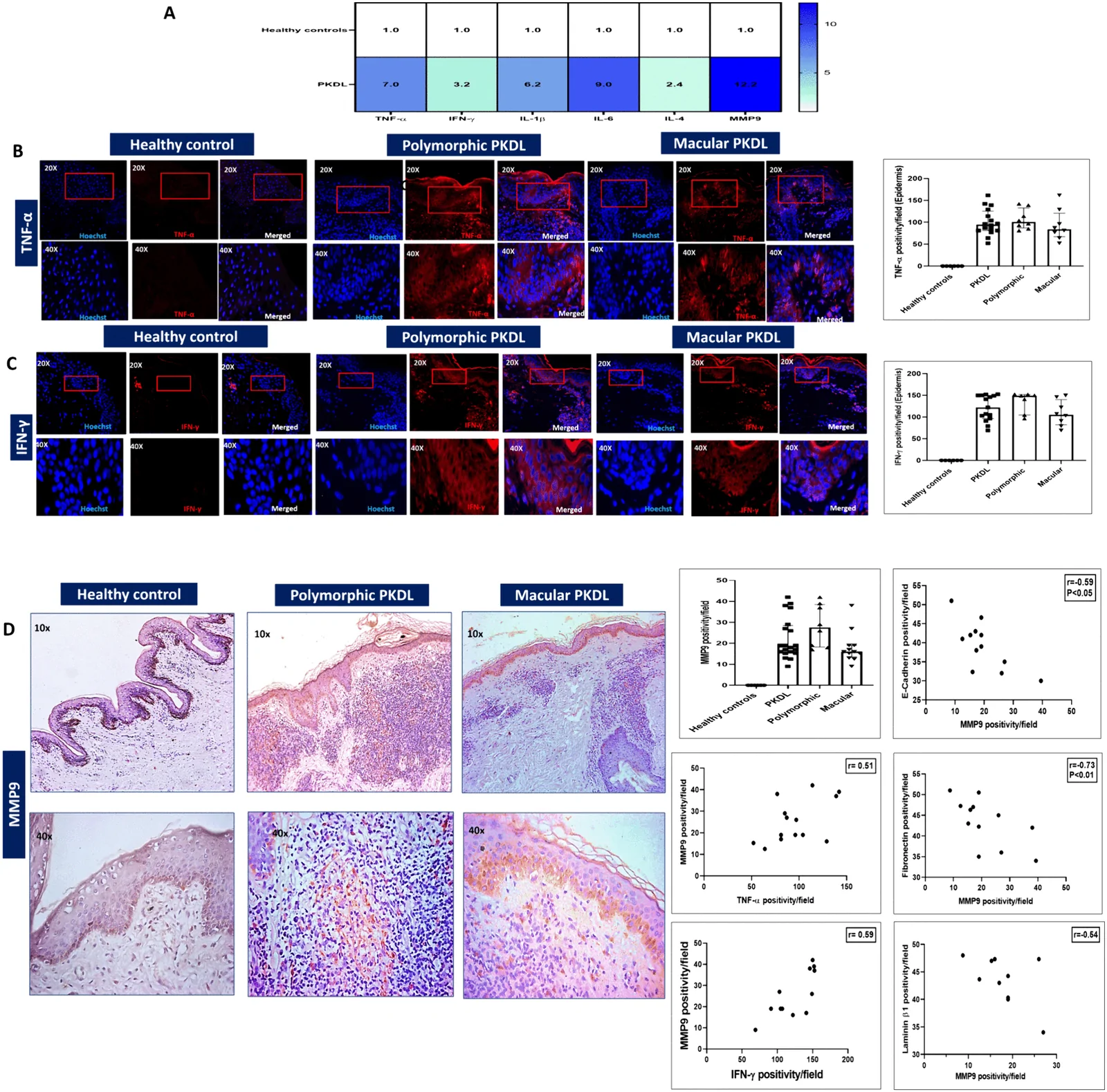
Dermal status of cytokines and MMP9 in patients with PKDL. **A:** Heat map indicating the fold change increase in levels of circulatory levels of cytokine milieu and MMP9 in patients with PKDL (n = 16) with respect to healthy controls (n = 9). The colour bar represents the range of fold change (1-13), with dark blue representing the highest fold change and white being the baseline. **B:** Representative immunofluorescence profiles of TNF-α expression from dermal biopsies of a healthy control, patient with polymorphic or macular PKDL (20x and 40x magnification), along with bar scatter plots showing the expression of TNF-α in healthy controls (●, n = 6), patients with PKDL (■, n = 16), either polymorphic (▲, n = 8) or macular (▼, n = 8) PKDL; each horizontal bar represents the median (IQR) fluorescence positivity/field. **C:** Representative immunofluorescence profiles of IFN-γ expression from dermal biopsies of a healthy control, patient with polymorphic and macular PKDL (20x and 40x magnification) along with bar scatter plots indicating the expression of IFN-γ in healthy controls (●, n = 6), patients with PKDL (■, n = 15), either polymorphic (▲, n = 7) or macular (▼, n = 8) PKDL; each horizontal bar represents the median (IQR) fluorescence positivity/field. **D:** Representative immunohistochemical profiles of MMP9 in dermal biopsies of a healthy control, patient with polymorphic or macular PKDL (10x and 40x magnification). Bar scatter plot demonstrating the status of MMP9 in healthy controls (●, n = 6), patients with PKDL (■, n = 20), either polymorphic (▲, n = 8) or macular (▼, n = 12) PKDL; each horizontal bar represents the median (IQR), along with correlation of MMP9 with lesional TNF-α (n = 14), IFN-γ (n = 12), E-Cadherin (n = 12), fibronectin (n = 12) and laminin (n = 10) fluorescence positivity/field.

At the lesional sites of PKDL cases, TNF-α and IFN-γ was minimally present in healthy controls [Fig 4B and 4C], whereas in PKDL lesions, TNF-α positivity was 94.5 (81.0-125.3) fluorescence positive cells/field, with a higher presence in polymorphic [100.5 (87.0-132.8) fluorescence positive cells/field], [Fig 4B] than macular PKDL, [84.0 (67.2-120.8) fluorescence positive cells/field], [Fig 4B]. Likewise, IFN-γ positivity in PKDL lesions was increased [122.0 (96.6-149.8) fluorescence positive cells/field], with polymorphic lesions showing a higher presence than macular lesions being [149.1 (104.8-152.0) and [105.0 (82.0-140.0) fluorescence positive cells/field] respectively [Fig 4C].

### Status of MMP9 in patients with PKDL

In vitiligo, the enhanced presence of MMP9 has been demonstrated to play a key role in degrading the tissue microarchitecture [8]. The circulating levels of MMP9, in PKDL cases as compared to healthy controls was increased by 12.2-fold, [1441500(1131675–1609125) vs. 117900(57750–291255) pg/ml, p < 0.0001, Fig 4D]. As compared to healthy skin where a minimal presence of MMP9 was observed, it was substantially increased in PKDL being [18.5(15.5-28.5)% positive cells/field], [Fig 4D]; this increased presence was observed in polymorphic and macular forms, being [27.5 (18.3-38.5) and 16.0 (13.5-19.0) percent positive cells/ field] respectively [Fig 4D]. In addition, a significant positive correlation was observed between lesional MMP9 positivity vs. TNF-α and IFN-γ, being r = 0.51, and 0.69, respectively [Fig 4D], whereas with cell adhesion molecule E-Cadherin, a significant negative correlation was present, [r = -0.59, p < 0.05; Fig 4D] as also with ECM components, fibronectin [r = -0.73, p < 0.01; Fig 4D] and laminin [r = -0.54; Fig 4D].

## Discussion

Leishmaniases encompass a diverse spectrum of clinical manifestations, primarily affecting the skin and mucosal surfaces, with varying degrees of tissue involvement and immunological responses. A consistent histopathological feature of all types of dermal leishmaniasis includes infiltration of immune cells. In CL, caused by *L. major,* a dense, diffuse inflammatory dermal infiltrate, predominantly composed of lymphocytes, histiocytes (macrophages), and plasma cells has been reported, along with a localized granulomatous immune response, perhaps aimed at parasite containment [33]. Similarly, in DCL, caused by *L.amazonensis/L.guyanensis* there is epidermal hyperplasia, and a diffuse dermal infiltrate predominantly composed of parasitized histiocytes, lymphocytes, plasma cells and multinucleated giant cells [33,34], with notably, no ulceration. Another non-ulcerated form of dermal leishmaniasis is the disseminated form of leishmaniasis (DL), that presents with a diffuse inflammatory dermal infiltrate mainly consisting of histiocytes, lymphocytes, and plasma cells [34,35]. PKDL, caused by *L. donovani* belongs to this group of non-ulcerated form of dermal leishmaniasis, along with features of hyperkeratosis, papillomatosis, and epidermal atrophy [3]. In the polymorphic form, the dense diffuse inflammatory infiltrate contrasted with the patchy perivascular infiltration in the macular form [Fig 1A] [3,16]. MCL shares the common feature of an inflammatory infiltrate that is dominated by neutrophils> CD4^+^ and CD8^+^ T lymphocytes> plasma cells> histiocytes, that are usually present in the mucosal lesions surrounding necrotic and perinecrotic areas [36].

CD68^+^ monocytes/macrophages constituted the predominant cell within the dermal infiltrate possibly because they serve as cellular niches that harbor the parasites [28,37,38]. In PKDL, the lesional CD8^+^ T cells are reported to be functionally anergic, and it was attributed to the IL-10-rich dermal milieu secreted by Foxp3^+^, CD25^+^, and CTLA-4^+^ nTreg cells. This immunosuppressive milieu could facilitate the establishment of a pro-parasitic microenvironment at the lesional sites [38–40]; a similar functional profile was reported in DCL [41]. The CD4 ⁺ Th1 cells are critical for protective immunity, while CD8 ⁺ T cells contribute both to parasite control and tissue damage, particularly through cytotoxic mechanisms as reported in CL and MCL [42]. Another important group are neutrophils, considered as Trojan horses for *Leishmania* parasites as they facilitate the transfer of internalized parasites to macrophages, thereby aiding in parasite dissemination [43,44]. In human CL and MCL, neutrophils have the MPO ⁺ /MMP9 ⁺ phenotype and this activated state possibly facilitates parasite killing, but as a collateral effect also drives tissue degradation resulting in ulcer formation [36]. The high proportion of CD20^+^ B cells and raised IgG levels [18] can facilitate parasite persistence and promote disease progression [Fig 1B-1F]. In terms of PKDL, this dynamic interplay between the parasite and host immune system translates into an altered inflammatory infiltrate, as the host mounts a parasiticidal response.

Chemokines are small signalling proteins that orchestrate the trafficking and localization of immune cells during homeostatic and inflammatory conditions. In CL, the lesions are marked by a high intralesional transcript level of CCL2 (MCP-1) that facilitates the migration of monocyte-macrophages. Additionally, an enhanced presence of CXCL9 (MIG), CXCL10 (IP-10), CCL3 (MIP-1α) and CCL5 (RANTES) ensures recruitment of Th1/Tc1 effector T cells, culminating in parasite control [45,46]. However, the increased expression of CCL3 mediates the pro-parasitic Th2 homing to lesions [47]. Similarly, in MCL, the increased presence of CXCL10 (and often CXCL9), ensured the recruitment of CXCR3 ⁺ Th1 and cytotoxic T cells [48]; In PKDL cases, the Ki-67 negative status along with the elevated levels of chemokines [Table 2], especially CCL2 facilitated the infiltration of monocytes-macrophages [3], while CXCL9/10, CCL3/4/5 possibly supported the recruitment of T and B cells [Table 2]. In autoimmune and inflammatory skin disorders, e.g., vitiligo, a prototype of hypopigmentation, CXCL9 and CXCL10 promote the dermal migration of cytotoxic CD8^+^ T cells, and possibly mediate melanocyte destruction [49]. Considering the overwhelming presence of CD8^+^ T cells in PKDL, one is tempted to propose a similar scenario accounted for the hypopigmentation observed in macular PKDL. However, as the CD8^+^ T cells in PKDL exhibit features of exhaustion [39], it remains an open ended question whether the CD8^+^ T cells facilitate hypopigmentation. Another important immune player is neutrophils based on the substantial presence of CXCL8 and their activated status [16], may also contribute towards the loss of melanin in macular PKDL [Table 2]. A limitation of this study is that the chemokines were measured in circulation, whereas the immune cell subsets were analysed in dermal lesions. Taken together, these findings are consistent with a potential role of these chemokines in the homing of immune cells to PKDL lesions.

Human skin consists of two main layers, epidermis and dermis, separated by the basal lamina and play a vital role in maintaining tissue architecture [50]. This interface contains keratinocytes and melanocytes connected via E-cadherin, and is essential for maintaining epidermal integrity and supporting the epidermal barrier function [51,52]. In vitiligo, the loss of E-cadherin has been linked to melanocyte detachment and hypopigmentation [8], and was endorsed in this study [Fig 2B].

ICAM-1 present on endothelial, epithelial, and immune cells, plays a pivotal role in leukocyte transendothelial migration, by facilitating leukocyte rolling, and directing their movement across the endothelium. Inflammatory stimuli such as TNF-α and IL-1β upregulate ICAM-1, and promotes migration of leukocytes via its binding to Leukocyte Function Antigen-1 [27,53]. In Sudanese PKDL, an elevated expression of ICAM-1 was reported [54], and was in concordance with the South Asian form [Fig 2B]. Importantly, its diffuse, perivascular distribution and positive correlation with the cellular infiltrate [Table 3], suggested a similar mechanism may contribute to the homing of immune cells to PKDL lesions. Other adhesion molecules such as VCAM-1 and selectins were not evaluated in the current work, but it would be worthwhile to study the same.

In healthy skin, the ECM ensures structural integrity and regulates cellular functions, key components being (a) fibronectin, that mediates an intricate fibrillar network at the cell surface, coordinating a range of cellular processes and supporting tissue architecture (b) collagen I and (c) collagen IV that provide a structural framework and (d) laminin that anchors epithelial cells and organizes the basement membrane [6]. The ECM not only offers structural and mechanical support, it also plays a crucial role in regulating melanocyte function [55]. In vitiligo, reduction in fibronectin, collagen IV, and laminin, was proposed to impact on survival of melanocytes by disrupting their attachment, resulting in hypopigmentation [9]. As hypopigmention is a consistent feature of South Asian PKDL [19], the decreased collagen I at lesional sites, [16] along with disruption of the ECM was further substantiated in this study by the decrease in fibronectin, collagen I, IV, and laminin [Fig 3A–3D]. Furthermore, this weakened structural integrity possibly facilitated trafficking of immune cells [Table 3] particularly host macrophages, allowing for decrease chronicity.

In dermal leishmaniasis, cytokine profiles within ulcerative and non-ulcerative lesions reflected distinct immune microenvironments, as for, e.g., in self-healing CL, the mixed Th1/Th2 response with a predominance of Th1 cytokines, IFN-γ, TNF-α, IL-12, IL-4, and IL-1, accounted for the parasite control, but also caused collateral tissue damage [48]. Similarly, in PKDL, the elevation of pro-inflammatory cytokines (TNF-α, IFN-γ, IL-1β, IL-6), anti-inflammatory cytokine IL-4, and immunomodulatory cytokines, TGF-β and IL-10, collectively accounted for the disease chronicity [18,35,44]. TNF-α disrupts melanocyte function by downregulating MITF, a key regulator of pigmentation, while IFN-γ enhances immune-mediated melanocyte destruction. These cytokines are implicated in classical hypopigmentary disorders such as vitiligo and post-inflammatory hypopigmentation [56], and their enhanced expression in PKDL lesions stand possible testimony of their ability to facilitate loss of melanin [Fig 4].

A key factor that destabilizes the tissue architecture as observed in vitiligo is the increased presence of MMP-9, which in the skin can be secreted by non-immune (e.g., keratinocytes, fibroblasts) or immune (e.g., neutrophils and macrophages) cells, and have been implicated in mucosal damage [36]. In vitiligo, the elevated levels of TNF-α and IFN-γ at lesional sites is proposed to trigger the secretion of MMP9, which causes degradation of E-cadherin and other ECM components, culminating in site-specific loss of melanocytes [8]. A similar scenario occurs in PKDL, where an elevated presence of TNF-α, IFN-γ and MMP9 was present at lesional sites and in circulation [Fig 3A and Table 4], endorsing that as in vitiligo, TNF-α and IFN-γ possibly enhanced MMP9 secretion and thereby reshaped the dermal architecture [Fig 3B–3D]. Notably, TNF-α and IFN-γ were mainly localized in the epidermis, while MMP9 was concentrated in the dermis and just below the basal layer, areas linked to hypopigmentation.

Taken together, the chronicity of PKDL was associated with a dermal infiltration of immune cells that included CD8^+^ T cells, CD68^+^ macrophages, CD20^+^ B cells, and CD66b^+^ neutrophils. The enhanced chemokine milieu possibly facilitated recruitment and retention of these immune cells within the lesions [57]. Furthermore, the elevated expression of pro-inflammatory cytokines, especially TNF-α and IFN-γ possibly promoted the secretion of MMP9, which in turn could contribute towards the degradation of the cell adhesion molecule E-cadherin and extracellular matrix components, e.g., fibronectin, collagen I, collagen IV, and laminin. This resultant alteration of these matrix components could facilitate immune cell infiltration and cause tissue remodeling, which could account for the clinical features associated with PKDL, e.g., hypopigmentation. However, in the absence of an animal model, direct evidence is difficult to establish. Collectively, these findings suggest that persistent immune dysregulation drives microarchitectural alterations in PKDL lesions, which can contribute towards the altered tissue pathology.

Owing to the social stigma and ostracization associated with PKDL, the mechanisms underlying hypopigmentation in PKDL do need to be delineated for improved treatment measures to be considered. As the present study has provided insights into tissue alterations associated with PKDL lesions and their possible role in facilitating hypopigmentation, the study holds public health relevance. A major limitation was the lack of age- and sex-matched healthy individuals for biopsy-based analyses, as these factors may influence extracellular matrix composition and tissue architecture. However, quantitative assessment showed no major differences in the cellular infiltrate or collagen I and IV (S1 and S2 Figs) the use of preputial skin as a feasible adult healthy-skin control. Ideally, the study should have also assessed the status of E-cadherin, ICAM-1, Fibronectin and laminin in preputial skin and is a limitation that needs to be addressed.

## Methods

### Ethical statement

The study was approved by the Institutional Ethics committee of Institute of Postgraduate Medical Education & Research & Seth Sukhlal Karnani Memorial Hospital [IPGME&R/IEC/2023/1051 Dated 06/12/23], and written informed consent was obtained from the individual or their legally accepted representative.

### Study population

The study population included archived biological material and freshly collected biological samples (plasma and formalin fixed paraffin embedded skin biopsies, FFPE), sourced from patients diagnosed with PKDL (n = 36), following passive surveillance (Dermatology Outpatient Department, College of Medicine and Sagore Datta Hospital, Kolkata (n = 6, 2022-date), or by active surveillance (n = 30, 2018-date) conducted in VL endemic districts of West Bengal, Malda (n = 20), Dakshin Dinajpur (n = 9), and non-endemic districts, e.g., Burdwan (n = 2) and Nadia (n = 1), along with Bihar (n = 3) and Jharkhand (n = 1, 2017-date) [3]. Using standard case definitions [World Health Organization. Leishmaniasis 2023. https://www.who.int/news-room/fact-sheets/detail/leishmaniasis/ accessed on 12th July, 2025], cases with a history of VL, presenting with skin lesions (presence of papules, macules and/or nodules) and rK39 positivity, were selected, and in case of archival samples, details were collected from the archived case report form. A dermal biopsy (3/4 mm) was collected from macules for the macular form (n = 13) and from nodules for the polymorphic form (n = 13), avoiding whenever possible aesthetically sensitive areas (e.g., face). The diagnosis of PKDL was confirmed by qPCR [20]. As a control group, skin from males undergoing circumcision (n = 12) or peripheral blood (age, gender matched, n = 11) was obtained [19]. Previous studies have confirmed that the histological architecture of preputial skin closely resembles adult skin, supporting its use as an appropriate healthy control [19, S1 and S2 Figs]. Peripheral blood (5.0 ml) was collected in heparinized vials (n = 11), and plasma stored at -80°C for further use. As PKDL is close to the point of elimination, the availability of patients was challenging; accordingly, calculation of power and sample size was not attempted.

### Reagents

All reagents were obtained from Sigma Aldrich (St. Louis, MO, USA) except anti-human E-Cadherin (clone NCH-38), CD8 (clone C6/144B), CD68 (clone PG-M1) [Dako, CA, USA], CD20 (clone L26), MMP 9 (clone EP127) [BioSB, Santa Barbara, CA, USA], anti-human ICAM-1, TNF-α, IFN-γ [BD Biosciences, San Jose, CA, USA], Fibronectin (FN1, A12392), Lamininβ1 (LAMB1, A9827), Collagen IV (Col4A3, A16359), Collagen I (Col1A1, A1352) and Goat anti-rabbit secondary antibody IgG-FITC (AS011) [Abclonal, Woburn, USA], antifadent [Southern Biotech, Birmingham, AL, USA], EnVision FLEX Target Retrieval Solution [Dako, CA, USA] and secondary detection system [DAB + chromogen, ScyTek Laboratories, West Logan, UT, USA], MMP9 Human ELISA Kit (RayBiotech, GA, USA), Bio-Plex Pro Human Chemokine Panel 40-Plex and 48-plex (for IFN-γ and TNF-α, BioRad, CA, USA).

### Histopathology

Formalin fixed paraffin embedded (FFPE) tissues were sectioned (3 μm), placed on poly L-lysine coated slides and examined by Hematoxylin and Eosin (H&E). Five randomly selected fields were manually counted under a light microscope (Carl Zeiss, Germany, Europe) at 40X magnification and analyzed using Q path 0.4.2. software (version 0.4.2, Belfast, Ireland) [16].

Sections (FFPE) were deparaffinised in xylene and rehydrated using descending grades of alcohol (100–70%) and distilled water. After heat induced epitope retrieval at pH 9 (high pH buffer) or at pH 6 (low pH buffer), the slides were incubated for 1 hr at room temperature (RT) with appropriate dilutions of primary antibody CD8 (1:25), CD20 (ready to use), CD68 (1:50) and E-cadherin, MMP9 (ready to use). The slides were then washed with Tris-buffered saline containing 0.05% Tween-20 (0.02M, pH 7.4, TBS-T), incubated with anti-polyvalent HRP polymer at RT for 1 hr and finally stained with DAB+ chromogen (1:100) at RT for 15 minutes as per the manufacturer’s protocol, and counter stained with haematoxylin.

DAB-positive cells appeared brown and were analyzed using Q path 0.4.2. software. To minimize bias, two blinded investigators independently evaluated the slides. For the polymorphic form, five randomly selected fields were assessed for cell distribution, whereas for macular lesions, cell enriched fields were counted under a light microscope (Carl Zeiss, Germany, Europe) at 40X magnification, averages taken and expressed as positive cells per field.

Immunofluorescence staining was performed on FFPE skin biopsies on Poly-L-lysine coated slides that were deparaffinized in xylene followed by rehydration in graded alcohol. The slides were incubated with NH_4_Cl (5 mM) for 20 min. to reduce autofluorescence. For antigen retrieval, slides were placed in a pre-warmed antigen retrieval solution (citrate buffer, pH 6.0) and incubated for 20 minutes. The slides were then brought to RT (approximately 30 minutes) and washed with phosphate buffered saline (PBS, 0.02M). After blocking the non-specific binding sites with PBS + 3% bovine serum albumin + 5% fetal bovine serum for 45 minutes, they were incubated overnight at 4°C with fluorescent conjugated anti- human fibronectin, collagen I, IV, laminin, ICAM-1-PE, CD66b-FITC, TNF-α and IFN-γ (diluted 1:50 in blocking buffer). Slides were washed thrice with PBS-Tween 20 (0.01% PBS-T) for 1 hr followed by an incubation with goat anti-rabbit secondary antibody IgG-FITC (1:200). Following three washes with PBS-T (20 minutes each) and a final wash with PBS for 10 min., they were counterstained with Hoechst (1 μg/mL, 50 μL, 10 min.), mounted with anti-fadent and stored at -20°C until imaging. The images were captured in both 20X and 40X using a Thunder imaging system (Leica, Wetzlar, Germany) and analyzed via Q path 0.4.2. software [16].

### Imaging analysis

Imaging for histopathology was conducted using a light microscope (Carl Zeiss, Germany, Europe) at 10x and 40x magnification. Tissue quantification (H&E, IHC, IF) was performed using QuPath (Quantitative Pathology), an open-source image analysis software (version 0.4.2, Belfast, Ireland). Total cellular infiltration was assessed by counting cells in five fields at 40x magnification, averaging the values, and expressing the results as the total number of positive cells per field. In H&E staining, each hematoxylin-positive cell nucleus was considered as a ‘positive cell,’ while in IHC/IF, chromogen/fluorescence-positive cells with hematoxylin/Hoechst-counterstained nuclei were classified as ‘positive cells’.

As all fields did not have expression of all immune cells studied, fields were selected on the basis of the presence of the marker being quantified. For markers with diffuse expression (e.g., MMP9, E-cadherin), percent positivity was determined. Immunofluorescence of ICAM-1, collagen I, collagen IV, laminin fibronectin, TNF-α and IFN-γ was assessed based on mean fluorescence intensity, as individual fluorescence/cell was not possible due to diffuse staining. The intensity was quantified as the amount of positive fluorescence per field at 40X magnification using QuPath. All analyses with Qu Path were conducted on ‘merged’ images. Immunofluorescence imaging was performed using Thunder imaging systems (Leica Microsystems, WZ, Germany) at 20x and 40x magnification, respectively. To reduce bias, two blinded investigators independently evaluated the slides.

### Quantification of circulating cytokines and Matrix metalloproteinase 9

Circulating levels of IFN-γ and TNF-α were assessed using a multiplex detection kit. Samples were diluted at a 1:4 ratio in sample diluent, as per the manufacturer’s instructions. Data acquisition was carried out on a Luminex 200 Labmap system (Luminex, TX, USA) and analyzed using BioPlex Manager software version 6.2. the concentrations of these cytokines were determined by interpolation from a standard curve. To ensure reproducibility, an internal control was included in all analyses. Additionally, circulating levels of MMP9 was quantified using a commercial kit (RayBiotech (GA, USA), samples were diluted 1:4 and processed as per the manufacturer’s protocol. Absorbances were measured at 450 nm using an ELISA reader (Merilyzer EIAQuant, Meril Life Sciences, India), the detection sensitivity being 74.07 pg/ml [16].

### Measurement of parasite load by real time PCR (rtPCR)

To measure parasite load, a standard curve was generated by adding a defined number of *Leishmania* parasites (ranging from 10 to 1 × 10^5^) from an *L. donovani* strain (MHOM/IN/83/AG83) to 180 μL of blood from a healthy control. Real-time PCR was conducted, template DNA being 0.1 μg and data expressed as parasites/μg of genomic DNA following extrapolation from a standard curve. In the polymorphic PKDL cases, the parasite load was usually quantified using a biopsy collected from nodular lesions, whereas in the macular PKDL group, biopsy samples were obtained from hypopigmented lesions.

### Statistical analysis

Data analysis between groups with non-parametric data was conducted using the Kruskal-Wallis’s test followed by Dunn’s multiple comparison test and expressed as median (IQR), or for parametric data, the one-way ANOVA followed by Tukey’s post hoc test and expressed as mean ± SEM. Correlations were assessed using Pearson’s or Spearman’s rank correlation for parametric and non-parametric data, respectively; a correlation coefficient (r) ≥ ± 0.4 was considered relevant. Statistical analyses were performed using GraphPad Prism (version 8.0.1, GraphPad Software Inc.), with p < 0.05 considered as statistically significant.

## Supporting information

S1 Data The minimal data set includes all raw data utilized in the statistical analyses presented in the figures, ensuring transparency and reproducibility of the results. (XLSX)

S1 Fig Status of the cellular infiltrate in preputial skin and adult healthy skin. **A, B**: Representative H & E profiles of the cellular infiltrate in dermal biopsy of a preputial skin (**A**) and adult healthy skin collected from Plastic and Reconstructive surgery (**B**).
(TIF)

S2 Fig Status of extracellular matrix components Collagen 1 and 4 in preputial skin and adult healthy skin. **A:** Representative immunofluorescence profiles of Collagen 1 expression in dermal biopsy of preputial skin (20x, **i**) and 40x (**ii**) magnification along with adult healthy skin collected from Plastic and reconstructive surgery (20x, **iii**) and 40x (**iv**) magnification: Representative immunofluorescence profiles of Collagen 4 expression in dermal biopsy of preputial skin (20x, **i**) and 40x (**ii**) magnification along with adult healthy skin collected from Plastic and reconstructive surgery (20x, **iii**) and 40x (**iv**) magnification).
(TIF)

S3 Fig Status of circulating chemokines and cytokines in patients with PKDL. **A, B**: Representative Whisker plots indicating status of circulating chemokines (**A**) and cytokines (**B**) in patients with PKDL.
(TIF)
